# Mechanical Integrity Degradation and Control of All-Solid-State Lithium Battery with Physical Aging Poly (Vinyl Alcohol)-Based Electrolyte

**DOI:** 10.3390/polym12091886

**Published:** 2020-08-21

**Authors:** Yaolong He, Shufeng Li, Sihao Zhou, Hongjiu Hu

**Affiliations:** 1 Shanghai Institute of Applied Mathematics and Mechanics, School of Mechanics and Engineering Science, Shanghai University, Shanghai 200072, China; yaolonghe@shu.edu.cn (Y.H.); lishufeng@shu.edu.cn (S.L.); zhoushihao9370@foxmail.com (S.Z.); 2 Shanghai Key Laboratory of Mechanics in Energy Engineering, Shanghai 200072, China

**Keywords:** solid polymer electrolyte, physical aging, viscoelastic, mechanical integrity, all-solid-state battery

## Abstract

Ensuring the material durability of an electrolyte is a prerequisite for the long-term service of all-solid-state batteries (ASSBs). Herein, to investigate the mechanical integrity of a solid polymer electrolyte (SPE) in an ASSB upon electrochemical operation, we have implemented a sequence of quasi-static uniaxial tension and stress relaxation tests on a lithium perchlorate-doped poly (vinyl alcohol) electrolyte, and then discussed the viscoelastic behavior as well as the strength of SPE film during the physical aging process. On this basis, a continuum electrochemical-mechanical model is established to evaluate the stress evolution and mechanical detriment of aging electrolytes in an ASSB at a discharge state. It is found that the measured elastic modulus, yield stress, and characteristic relaxation time boost with the prolonged aging time. Meanwhile, the shape factor for the classical time-decay equation and the tensile rupture strength are independent of the aging history. Accordingly, the momentary relaxation modulus can be predicted in terms of the time–aging time superposition principle. Furthermore, the peak tensile stress in SPE film for the full discharged ASSB will significantly increase as the aging proceeds due to the stiffening of the electrolyte composite. It may result in the structure failure of the cell system. However, this negative effect can be suppressed by the suggested method, which is given by a 2D map under different lithiation rates and relative thicknesses of the electrolyte. These findings can advance the knowledge of SPE degradation and provide insights into reliable all-solid-state electrochemical device applications.

## 1. Introduction

With the deepening research on all-solid-state batteries (ASSBs), an intensive scientific interest in the development of inherent safety, non-leakage, and stable high-performance electrochemical equipment has emerged to accommodate a wide range of new engineering applications, including medical implants, flexible electronics, and textiles [1,2]. In contrast to inorganic counterparts with a brittle crystalline phase, the solid polymer electrolyte (SPE) exhibits excellent interfacial compatibility, and superior elasticity to endure greater mechanical deformation, contributing to be cast into the complicated architectures for lithium-ion and lithium-metal batteries [3,4,5]. Consequently, a wide array of polymers such as poly (ethylene oxide) (PEO) [6,7,8,9], polyacrylonitrile (PAN) [10,11,12,13], poly (vinylidene fluoride) (PVdF) [14,15], and its copolymer with hexafluoropropylene (PVdF-HFP) [16,17], poly (methyl methacrylate) (PMMA) [18,19,20], and poly (vinyl alcohol) (PVA) [21,22,23,24], as well as their mixtures [25,26,27,28], have been adopted as the potential matrices for solid electrolytes. Especially, the outstanding inherent advantages (nontoxicity, biocompatibility, biodegradable, good mechanical strength, relatively high dielectric constant, and charge storage capacity) of PVA make it one of the preferred choices for such applications [29,30,31]. Actually, with the help of dimethyl phthalate (DMP) and lithium perchlorate (LiClO_4_), PVA-based SPEs exhibited an ionic conductivity of 0.149 × 10^−3^ S·cm^−1^ at room temperature [32]. In addition, the rich hydroxyl groups attached to the polymer chain of PVA also provide strong hydrogen binding, contributing to an excellent mechanical stability and high melting point [33]. However, due to a rapid solvent volatilization or the steep cooling from melting temperature in the process of solidification, all of above amorphous or semi-crystalline polymers are inevitably in the high nonequilibrium state. The electrolyte material with excess thermodynamic quantities would spontaneously evolve with time to equilibrium, which is referred to as physical aging [34,35], leading to pronounced variations in the microstructure and also macroscopic properties under storage and servicing operations. Accordingly, the physical aging process of SPEs and its effects on ASSB performance is a pivotal scientific problem to be solved.

Evolution of the aging time-dependent conductivity of SPEs is indispensable to predict the long-term life of an electrochemical apparatus accurately. Therefore, growing attentions have been paid to experimentally investigate the ionic conduction of polymer electrolyte materials and the potential relaxation mechanism during the physical aging. In the first place, it was reported that physical aging could cause a decrease in the size of the coordinating sphere around the cation. As a consequence, the conductive performance of PEO-LiClO_4_ enhanced as the storage time increased [36]. On the other hand, SPEs consisting of poly (acrylonitrile-*co*-butyl acrylate) and LiTFSI or LiI, as well as a LiTFSI salt mixture, exhibited a dropped conductivity with elapsed time because the continuity of conductivity pathways ground on ion–ion interactions had been damaged to some degree [37]. In addition, for the SPE based on the hybrid of PEO and PMMA with lithium triflate, the ionic conductivity was found to initially rise markedly, followed by a reduction by over one order of magnitude as the aging process extended up to one year [38]. These distinct experimental results imply that the time-aging process played a complicated role on the lithium-ion diffusion in solid ion-polymer complexes. It is still an open question how the evolution of the structure-related behavior of the electrolyte material subjected to the physical aging couples lithium salts.

As the mechanical failures of cell components are thought to be crucial factors for the capacity degradation of ASSB, a great deal of researchers have oriented their efforts toward clarifying the mechanical response and the potential endangerment mechanism of the cell systems in the recent three years [39,40,41,42,43,44]. One has gradually recognized that the Vegard stress induced by the electrochemical reaction at the cathode can not only imperil both active substances and bonding materials in the composite electrode, but also injure the solid electrolyte which acts as the separator sandwiched between the anode and cathode. For example, in terms of a fully coupled electro-chemo-mechanical model, Bucci et al. quantitatively explored the mechanical reliability of ASSB for the first time, and found that the deformation of lithium embedded in active particles could cause inorganic electrolyte fracture [44]. Notedly, even for the polymer electrolyte material, the stress magnitude was indeed high, and also resulted in the material degradation of SPEs during service [39]. On the other hand, the stress development in the inorganic solid electrolyte might also originate from the interfacial incompatibility of the electrode and electrolyte [43]. The crack initiation and propagation in the solid electrolyte for a Li/Li_1+x_Al_x_Ge_2−x_(PO_4_)_3_(LAGP)/Li cell were captured by in situ X-ray computed tomography under a charging–discharging cycle, and the fracture occurred in the interphase region between the electrolyte and electrode [45]. The simulation also demonstrated that lithium insertion would produce dendritic cracking in ceramic solid electrolytes [46]. Similarly, Herbert and co-workers reported that localized stress intensification at the lithium/solid electrolyte interface might contribute to the short-circuiting of ASSB [47]. Compared to extensive research on the internal stress in ASSB with inorganic solid electrolytes, very little literature has documented the mechanical-electrochemical behavior of cells based on SPEs. Although some progress has been achieved on the issue [39,40], the structure relaxation of electrolyte materials has not been taken into account. Up to now, major challenges remain in developing polymer electrolyte materials to meet the requirements of commercialization, and one of the pressing tasks is how to control the mechanical deterioration of ASSB with time-aging SPEs under the circumstance of usage.

The objective of this study is to disclose the physical aging and viscoelastic behavior of polymer electrolyte and further investigate its underlying impact on the mechanical stability of cell systems in service, and give the theoretical support for the optimal design and utilization of advanced secondary batteries. To achieve this goal, we add LiClO_4_ salt into PVA with a weight ratio of 40% to prepare the SPE film, and then carry out a series of quasi-static uniaxial tension and stress relaxation tests on the glassy electrolyte. Subsequently, the dependency of SPE viscoelasticity on elapsed time is discussed in detail. Following that, a continuum electrochemical-mechanical model is built to analyze the stress evolution and mechanical integrity of time-aging electrolytes in an ASSB upon various electrochemical rates and the relative thickness of SPE film to the cathode, respectively. Further, a feasible method for improving SPE integrity will also be proposed in the light of massive simulations. This work is expected to thoroughly understand the long-term mechanical responses of a polymer electrolyte composite and provide insights into durable energy conversion and storage devices.

## 2. Experimental

### 2.1. Materials

Poly (vinyl alcohol) (Mowiol^®^ PVA-203, Aladdin, Shanghai, China) particles with an alcoholysis degree of 86.7–88.7% were adopted as the matrix of solid electrolyte, while LiClO_4_ (Aladdin, Shanghai, China) had a weight fraction of 40% and used as the lithium salt for the electrolyte materials. Moreover, SPE films were prepared using the following procedure. First, PVA-203 was fully dissolved in deionized water with a mass ratio of 1:10 at 95 °C for 2 h under mechanical agitation at 90 r/min. Then, LiClO_4_ powders were added into the PVA aqueous solution, which was cooled at 50 °C, and entirely mixed for about 2 h. Secondly, the obtained PVA-LiClO_4_ aqueous solutions were slowly cooled to ambient temperature (28 °C), and then degassed for 30 s. Finally, they were casted onto leveled Teflon-coated glass plates, and then dried at 40 °C for 72 h in the vacuum oven. Dried SPE films with a thickness of 100 µm were cut into dimensions of 20 mm × 5 mm, annealed at 100 °C for 30 min to erase the previous thermal history, and then kept in a glove box with a protective argon atmosphere at 28 °C prior to all of the following experiments including thermogravimetric analysis (TGA), differential scanning calorimetry (DSC), and uniaxial tension, as well as stress relaxation.

**Figure 1:**
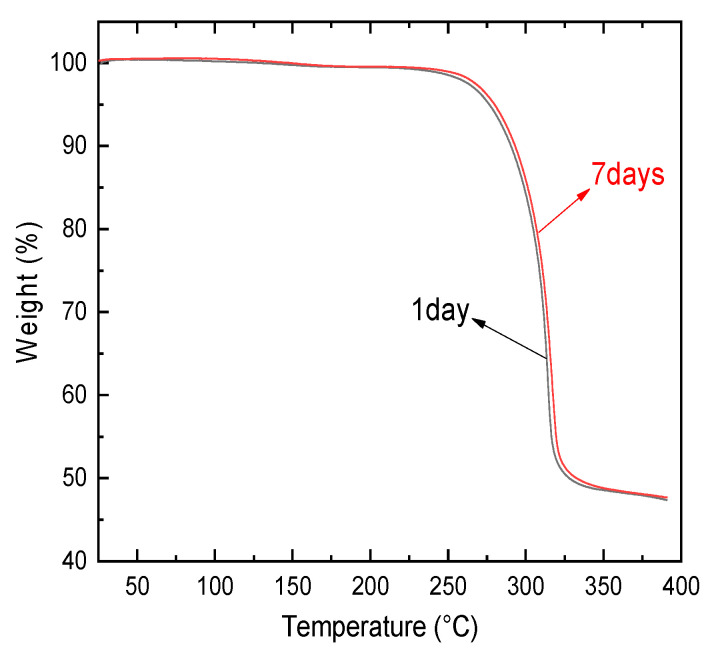
TGA of PVA-LiClO_4_ aged for different elapsed times.

In order to check whether a crystallinity change and phase separation of the aging SPE would occur in absorbed water, TGA and DSC experiments were respectively carried out for the PVA-LiClO_4_ specimen during the aging process. TGA was used by TA-Q500 (TA Instruments-Waters LLC, New Castle, DE, USA) in the temperature range from 28 °C to 400 °C at a scan rate of 10 °C/min under a nitrogen atmosphere. As can be seen in Figure 1, no weight loss is found below 100 °C, indicating that there is no water absorbed in the testing PVA-LiClO_4_ films aged for 1 or 7 days. That is to say, the residual water in SPE films had been eliminated with the help of the annealing step at 100 °C, and the specimen was kept well-dried under the storage environment.

**Figure 2:**
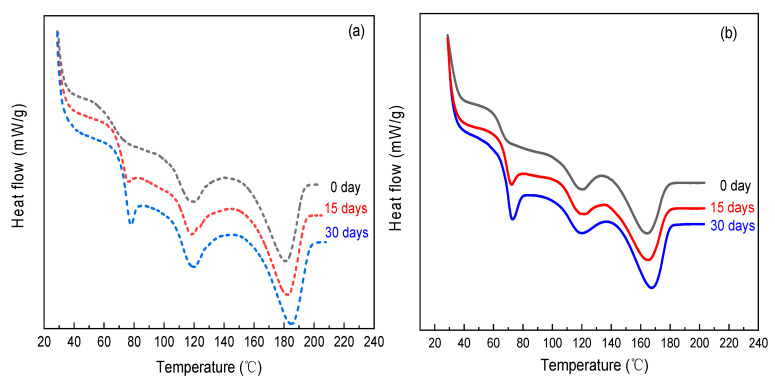
Effect of elapsed time on DSC thermograms of (**a**) PVA and (**b**) PVA-LiClO_4_.

The thermal behaviors of the pure PVA and LiClO_4_-doped specimens were measured using modulated DSC of TA-Q2000 (TA Instruments-Waters LLC, New Castle, DE, USA) in the temperature range from 28 °C to 210 °C at a scan rate of 30 °C/min under a highly purified nitrogen atmosphere. According to DSC heating thermograms of the PVA, as seen in Figure 2a, it is observed that the glass transition had occurred at 70 °C for the freshly quenched specimen (elapsed time: 0 day), and the peaks at around 120 °C and 181 °C correspond to the crystallization temperature ($T_{c}$) and melting temperature ($T_{m}$) of the semicrystalline polymer, respectively. Due to the decrease in free volume of the system during the annealing period, the values of $T_{c}$ and $T_{m}$, as well as glass transition temperature ($T_{g}$), tend to rise slightly with the elapsed time. Furthermore, PVA samples aged for 15 and 30 days exhibit an observable endothermic overheating peak near the glass transition region, which indicates the recovery of heat loss during the enthalpy of relaxation [48]. This peak area would increase with the physical aging, and the corresponding apex temperature is also found to slightly shift toward higher temperatures as a consequence of declining molecular mobility. Compared to pure PVA, a similar DSC heating thermogram is indicated in Figure 2b for the LiClO_4_-doped PVA complex except at lower $T_{g}$, $T_{m}$, and degree of crystallinity owing to the plasticization of lithium salts on PVA. For both PVA and PVA-LiClO_4_, the almost constant enthalpy of fusion means that the crystallinity may maintain stability as the elapsed time increased from 0 to 30 days. In other words, the phase separation did not take place under the aging condition.

### 2.2. Test Methods

Mechanical properties of SPE films upon various aging times were measured in terms of uniaxial tension by a DMA and DMA-RH accessory (Q800, TA Instruments-Waters LLC, New Castle, DE, USA) under a dried environment (RH < 5%) at ambient temperature (28 °C). The experiments were fulfilled at the elapsed time of 1, 5, 10, 20, and 30 days, which followed the snapshot assumption proposed by Struik [34].

(1) For the purpose of determining the tensile properties of SPE films in the period of physical aging, quasi-static uniaxial tension was conducted at the strain rate of 5 × 10^−3^ s^−1^.
(2) The stress relaxation was carried out at 0.4% strain, which could ensure that the mechanical response of the sample was within a linear range. The stress required to keep a constant strain was recorded with a sampling rate of 10 Hz.

## 3. Model Formulation

**Figure 3:**
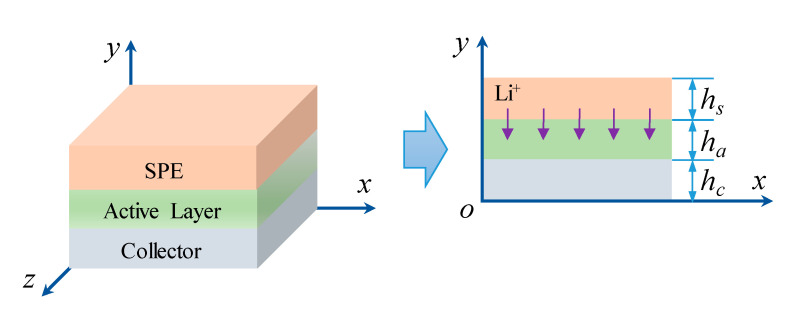
Schematic of the model geometry.

As solid polymer electrolytes in ASSB usually exhibit the coupling behavior of electrochemistry and mechanics, the issue that specifies the cell system accounting for the electrolyte relaxation can become more complicated. Herein, concentrating on exploring the evolution stress of aging SPEs and related mechanical integrity problems, we follow the recent work of Grazioli et al. [39] and consider a uniform planar half-cell comprising a multilayered SPE and cathode (active layer and corresponding current collector), as shown in Figure 3. The anode of the metal lithium layer is ignored because of its low modulus to simplify the model. Upon discharging or charging operations, lithium inserts into or extracts from the active material as a result of the electrochemical energy conversion, drives deformation within the structure, and thus leads to internal stress in the electrode and SPEs.

### 3.1. Migration of Lithium

Considering that lithium diffusion in the active layer obeys Fick’s law, the mass conservation describes the migration of lithium as

(1) $$\frac{\partial c_{\mathrm{Li}}}{\partial t}+\mathrm{div}J=0$$

where $J=D\nabla c_{\mathrm{Li}}$ is the flux of lithium and D is the related diffusion coefficient of lithium.

### 3.2. Internal Stress

Internal stress arises when the active particles inflate or deflate as a consequence of lithium migration. According to the literature [49], the additional strain occurring in this process can be analogous to the thermal strain and expressed as

(2) $$\varepsilon^{Li}=\frac{\Omega }{3}\left(c_{\mathrm{Li}}-c_{\mathrm{Li},\mathrm{init}}\right)I$$

where Ω is the partial molar volume of the host material, $\left(c_{\mathrm{Li}}-c_{\mathrm{Li},\mathrm{init}}\right)$ represents the change in lithium concentration relative to the initial state, and I is the identity tensor.

In the case of elastic electrode material, the total strain is the sum of the lithiation strain shown above in Equation (1) and the elastic strain caused by the structural deformation, i.e.,

(3) $$\varepsilon =\varepsilon^{e}+\varepsilon^{Li}$$

To specify the stress associated with the above lithium insertion or extraction, additional governing equations in solid mechanics should be introduced. First, the balance of force for any solid element without the action of body force in static equilibrium provides divσ=0. Then, considering that the deformation of cathode material would be small as the maximum swelling deformation investigated here is less than 10%, the strain tensor is thus defined by $\varepsilon =\left(\nabla u+\nabla u^{T}\right)/2$, where u is the displacement vector. In addition, to describe the relation between strain and stress, the constitutive equation should also be introduced. For an isotropic linear elastic medium, like the active layer and collector, we can write

(4) σ=2G devε−pI

Here, G is the shear modulus, and devε=ε−trε/3 I is the deviatoric component of the strain, where $\mathrm{tr}\varepsilon =\sum_{i}\varepsilon_{ii}$ is the related trace. The pressure (p) is equal to −trσ/3 and is proportional to the volume change of the material, namely, $p=-K\left[\mathrm{tr}\varepsilon -\Omega \left(c_{\mathrm{Li}}-c_{\mathrm{Li},\mathrm{init}}\right)\right]$, where K is the bulk modulus. Yet, for the viscoelastic SPEs, the first term on the right of Equation (4), i.e., the deviatoric stress (devσ), is not linearly related to the deviatoric strain (devε), but also depends on the strain history, which is normally defined by the hereditary integral as

(5) $$\mathrm{dev}\sigma =2\int_{0}^{t}\Gamma \left(t-t^{\prime }\right)\frac{\partial \mathrm{dev}\varepsilon }{\partial t^{\prime }}dt^{\prime }$$

Here, Γ(t) is the relaxation modulus function, and can be obtained by measuring the stress variation of SPEs against loading time at a constant strain.

### 3.3. Solving Conditions

To solve the elastic-viscoelastic problem hereinabove, proper initial and boundary conditions should also be determined. With regard to the lithium concentration, one may consider that the SPE is initially lithium-free, i.e., $c_{\mathrm{Li},\mathrm{init}}=0$, and then undergoes lithiation at a constant current (CC) until the maximum concentration is achieved at the inlet surface ($z=h_{c}+h_{a}$). Thereafter, it is set as constant to mimic the constant voltage (CV) operation and to make the electrode fully lithiated in the following electrochemical process. Thus, the corresponding initial and boundary conditions for lithium diffusion are

(6) $$\begin{array}{l} c_{\mathrm{Li}}=0\;\;\;\mathrm{at}\;\;\;t=0\\ -n{\cdot J|}_{z=h_{c}}=0,\;\;-n{\cdot J|}_{z=h_{c}+h_{a}}=\frac{i_{n}}{F}\;\;\;\mathrm{at}\;\mathrm{constant}\;\mathrm{current}\;\mathrm{stage}\\ -n{\cdot J|}_{z=h_{c}}=0,\;\;{c|}_{z=h_{c}+h_{a}}=c_{\max}\;\;\;\;\;\;\;\mathrm{at}\;\mathrm{constant}\;\mathrm{voltage}\;\mathrm{stage} \end{array}$$

where *F* is Faraday’s constant, and $i_{n}$ and $c_{\max}$ are the surface current density and stoichiometric maximum concentration of the solute atoms, respectively.

As for the solid mechanics simulation, the composite electrode is assumed stress-free at the initial state and then experiences the deformation as the active materials are lithiated. The SPE film is assumed to be a homogeneous material and perfectly bonded to the electrodes. This contributes to the continuity of the normal stress and displacement at the interfaces between each layer. As we focus on the electrolyte stress generated by the electrode exposed to lithium insertion, the outer surfaces of the structure are thus in traction-free conditions. In addition, side reactions are not taken into account in the simulations.

## 4. Results and Discussions

### 4.1. Mechanical Performance of SPEs during Physical Aging

**Figure 4:**
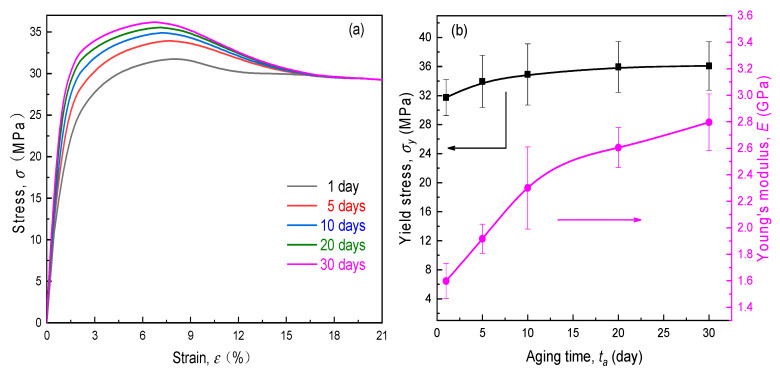
Effect of aging time on the (**a**) stress–strain curves and (**b**) tensile properties of solid polymer electrolyte (SPE) films.

The uniaxial stress–strain curves of PVA-LiClO_4_ films aged with various time durations are indicated in Figure 4a. It is observed that due to the plasticization of lithium salt on the semi-crystalline PVA, the tensile response of SPEs seemed to resemble that of a representatively ductile material, exhibiting a yield point before fracture happened. As one would expect, the tensile behavior of electrolyte material evolved with elapsed time ($t_{a}$). The impact of aging was not confined to the linear stage, but was also found in the nonlinear range. Further, an increasing aging time led to the rise in both Young’s modulus (E) and yield stress ($\sigma_{y}$), which can be ascribed to the decrease in the fraction of free volume in the aging SPEs. However, the influence on the value of $\sigma_{y}$ would gradually decrease as the physical aging proceeded (see Figure 4b). After an aging time of 20 days, the yield strength might level off and remain constant at around 36.2 MPa thereafter. Besides, the rupture stress appeared not to be dependent on the aging process as a result of the mechanical rejuvenation, i.e., the plastic deformation could erase the prior thermal history to a certain extent. Even so, the effect of physical aging should be taken into account due to the dramatic increase in the stiffness of the SPE film, as shown in Figure 4b, where the magnitude of E at the thirtieth day was nearly twice that for the unaged specimen. Therefore, the time-dependent-elastic modulus needs a more in-depth discussion as follows.

**Figure 5:**
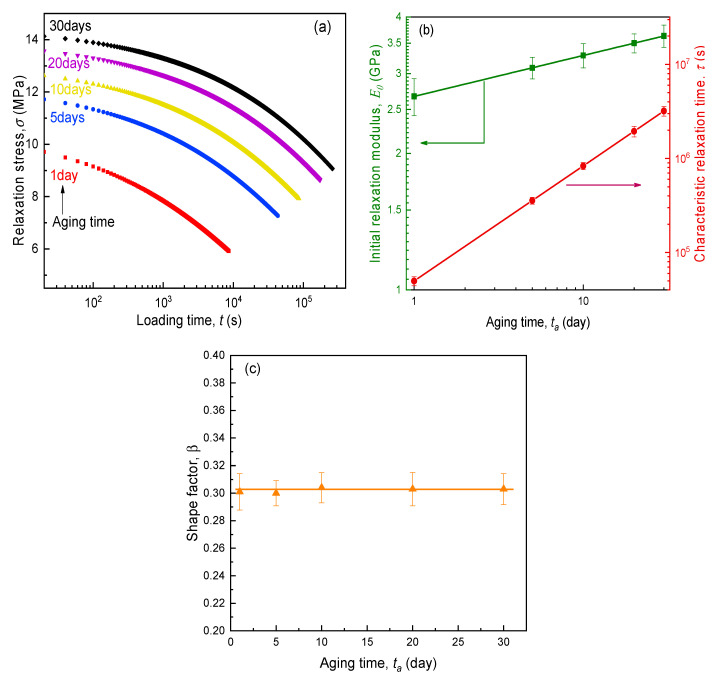
Effect of physical aging on (**a**) stress relaxation curves and Kohlrausch–Williams–Watts (KWW) parameters: (**b**) $E_{0}\;$ and τ, (**c**) β.

Under a constant small strain (0.4%), the variation in tensile stress (σ) against loading time (t) for the SPE film aged from 1 to 30 days is indicated in Figure 5a. All experimental curves of σ(t)−logt showed a profound time-related stress reduction, but with obviously different relaxation extents, implying that the physical aging acted upon an important role on the viscoelastic behavior of electrolyte composites. At each aging time, the stress magnitude reduced up to nearly half of the initial value (t=0) after the Struik loading time, i.e., $t=0.1t_{a}$ [34]. In addition, the earlier the aging stage, the more rapid the relaxation in stress. This thus demonstrated that the physical aging would slow down the relaxation process of SPE films. As present relaxation behaviors were in the range of linear viscoelasticity, the relaxation modulus $E\left(t\right)=\sigma \left(t\right)/\varepsilon_{0}$ was irrelevant to the strain level. Hence, the evolution of E(t) could be analyzed by the following Kohlrausch–Williams–Watts (KWW) equation for the purpose of elucidating the fundamental mechanism.

(7) $$E\left(t\right)=E_{0}\exp{\left(-\frac{t}{\tau }\right)}^{\beta }$$

where $E_{0}$ is the initial relaxation modulus (t=0), t is the loading time, τ is the characteristic relaxation time, and β is the shape factor for the relaxation curves.

**Figure 6:**
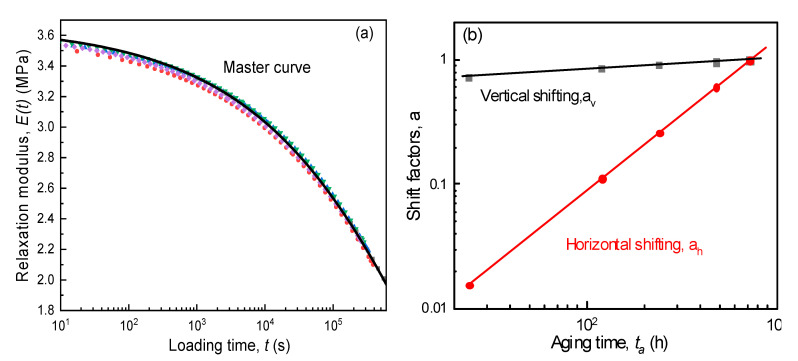
Relaxation master curve (**a**) and shift factors (**b**) for SPE films.

As expected, the regression correlation correlations are more than 0.99, meaning that relaxation modulus curves of SPE films during isothermal physical aging were fitted very well by the above KWW formula, and the evolutions of relaxation characteristics ($E_{0}$, τ, and β) are shown in Figure 5b,c. As observed from the KWW parameter curves, with increased aging time, the magnitudes of both $E_{0}$ and τ linearly ascended steeply in double logarithmic coordinates, while β kept a constant of 0.30. This suggests that all curves of E(t)−t for SPE films at various aging stages had a similar shape and could be superimposed by the Struik shift method [34], which was based on effective time theory. For convenience of processing data, the relaxation curve at the longest aging time (30 days) was selected as the reference, and the modulus data at other aging times were horizontally and vertically shifted until they completely overlapped the reference curve. The shifting results and associated horizontal and vertical shift factors given by Equations (8) and (9) are shown in Figure 6a,b, respectively. The smooth master curves indicate the validity of the time–aging time superposition principle, as seen Figure 6a. Moreover, it can be further verified from Figure 6b wherein both the horizontal and vertical shift factors linearly increased with aging time in double logarithmic coordinates. Therefore, as long as the loading time is less than or equal to one tenth of the aging time, one might obtain the predicted elastic modulus of SPEs in the process of physical aging according to the relaxation master curve and shift factor. As our longest aging test lasted for one month (720 h), it was obviously larger than ten times the electrochemical operation time of the lithium secondary battery. Hence, based on the data indicated in Figure 6, we deliberate the stress variation and mechanical integrity of the time-aging SPE in an ASSB in next sections.

(8) $$a_{h}=\frac{\tau (t_{a})}{\tau (t_{a}^{ref})}$$

(9) $$a_{v}=\frac{E_{0}(t_{a})}{E_{0}(t_{a}^{ref})}$$

where $a_{h}$ and $a_{v}$ are horizonal and vertical shift factors, respectively, and $t_{a}^{ref}$ is the reference aging time.

### 4.2. Impact of Physical Aging on SPE Stress during Electrode Lithiation

**Figure 7:**
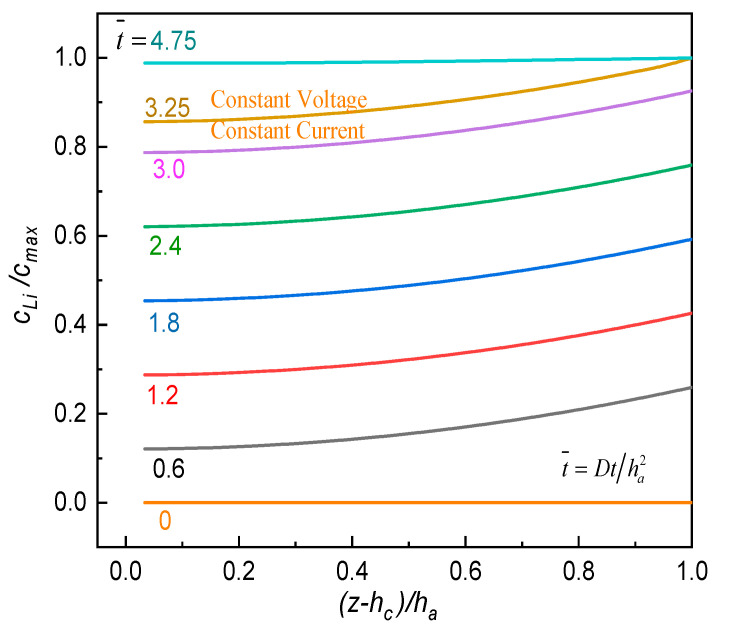
Lithium-ion profiles in active layer at different lithiation times.

**Figure 8:**
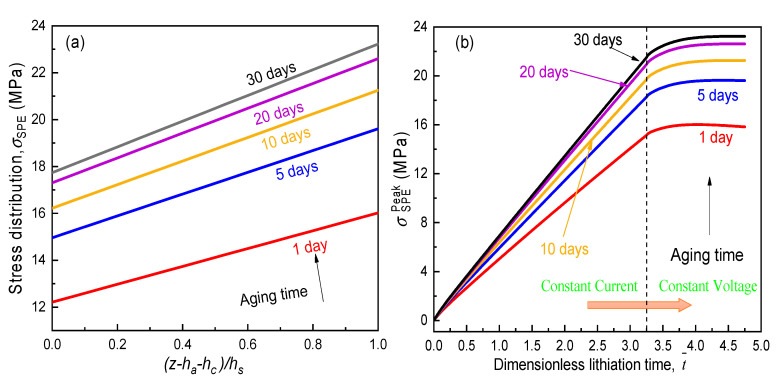
Effect of aging time on the (**a**) stress profile across the SPE and (**b**) peak SPE stress.

During the electrochemical operation of an ASSB, one recognizes that internal Vegard stress within the cell system is mainly induced by the swell or contraction of active layers. As the material properties of SPE films evolved as the aging time elapsed, we are very interested in whether their mechanical integrity in service can be threatened. Hence, Figure 7 and Figure 8a,b were prepared to show the evolution of lithium-ion concentration in the active layer and the stress profile as well as the peak stress within the SPE film, respectively. To obtain a maximum influence of the electrochemical loading on the solid electrolyte, the active materials were completely lithiated with a constant current−constant voltage operation through the method described in Section 3 above. The related model parameters are listed in Table 1.

The detailed lithiation process of the LiCoO_2_/PVdF/CB(carbon black) cathode is demonstrated in Figure 7. Initially, the electrode showed a uniform lithium-ion concentration of $c_{0}=0\;mol/m^{3}$. Subsequently, it was intercalated galvanostatically with a current rate of 1 C until the inlet surface $z=h_{a}$ attained saturation, where the normalized discharging time ($\bar{t}=Dt/h_{a}^{2}$) for this stage was about 3.25. Further, in order to make the composite electrode totally lithiated, potentiostatic operation by holding the surface concentration had been implemented before the active material near current collector was also full of lithium ions. Then, with the obtained distribution of lithium-ion concentration across the active layer, a set of viscoelastic solid mechanics problems considering physical aging were solved with the help of the finite element method. That is to say, the stress distribution in the aging SPE film for an ASSB at any lithiation could be obtained. Figure 8a tracks the stress profile across SPE thickness at the time of completed lithiation on the cathode. All the SPE stresses are found to be tensile and significantly increased as the aging advanced, and the maximum value occurred near the free surface of SPE. Furthermore, we are centered on the variation in the peak stress ($\sigma_{SPE}^{Peak}$) in the SPE film during the entire galvanostatic-potentiostatic (CC-CV) loading process against various aging times as depicted in Figure 8b. It can be seen that the magnitude of $\sigma_{SPE}^{Peak}$ raised rapidly at the CC stage while beginning to climb slowly at the CV operation. As anticipated, physical aging led to a dramatic enlargement in this peak stress level due to the stiffening of the electrolyte material. Although the negative effect gradually decreased with the elapsed time, $\sigma_{SPE}^{Peak}$ at the completely discharged state for the SPE film after 30 days of aging was about one and a half times that of the one-day-aged specimen. These findings indicate that physical aging of the SPE film may degrade the structure firmness of an ASSB and need to be controlled. Therefore, the potential design factors, including the geometric parameter and electrochemical conditions, are discussed in the following sections.

### 4.3. Impact of Relative Thickness of SPE Film

As an SPE film plays the dual role of both electrolyte and separator, on the one hand, it should be as thin as possible to reduce the resistance of lithium-ion transport. On the other hand, increasing the SPE thickness is beneficial to enhancing the safety index of the component. It is a primary problem how to rationally make a choice for the cell geometric parameter to meet a compromise in electrochemical performance and mechanical integrity of the electrolyte material during the aging process.

**Figure 9:**
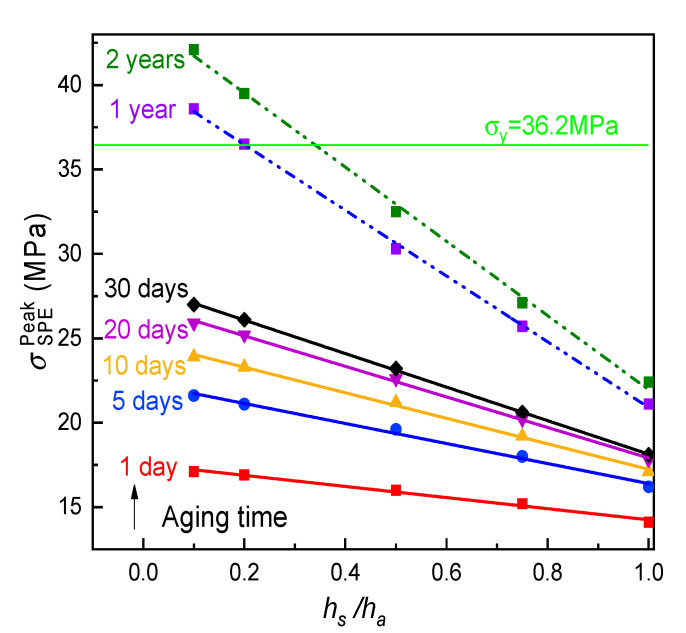
Effect of thickness ratio ($h_{s}/h_{a}$) on the peak tensile stress of SPE at various aging times.

Under the same electrochemical operation condition as Section 4.2 above, the great dependency of $\sigma_{SPE}^{Peak}$ on the thickness ratio of the SPE film to the active layer ($h_{s}/h_{a}$) is displayed in Figure 9. First, for the specimen aged after one day, this stress dropped nearly 20% as the relative thickness rose from 0.1 to 1.0. This is ascribed to the fact that an increase in the SPE thickness contributed to the stronger structural limitation on the lithiation expansion of the LiCoO_2_/PVdF/CB electrode due to the competitive modulus between the solid electrolyte to the composite cathode. Hence, the induced internal stress in the SPE/cathode interface, i.e., the magnitude of $\sigma_{SPE}^{Peak}$, could linearly lessen with $h_{s}/h_{a}$. Further, the stress inhibition resulting from the thicker SPE film would become more prominent with the physical aging process as a consequence of more significant deformation constraints caused by the hardening of the aging electrolyte. Second, at the same thickness ratio, the peak stress exhibited a non-linear growth trend with the aging. It is wondered whether the thinner and deeply aging electrolyte in service retains structural integrity or not. As seen in Figure 9, for the extremely thin SPE film ($h_{s}/h_{a}=0.1$), the value of $\sigma_{SPE}^{Peak}$ augmented up to 28.0 MPa after 30 days of aging. However, recalling the aforesaid mechanical testing results, the yield stress ($\sigma_{y}$) of SPE films at the aging time from 1 to 30 days was larger than 32.0 MPa, as shown in Figure 4b, suggesting that the yield would not occur for either thin or thick PVA-LiClO_4_ films in an ASSB among one month. Conversely, with regard to the longer aging time, this situation may change. In this case, one can optimize the cell geometries by controlling $h_{s}/h_{a}<\left[h_{s}/h_{a}\right]$, where $\left[h_{s}/h_{a}\right]$ is the critical thickness ratio at which aging SPE reaches its allowable stress such as the yield stress. On the basis of the fact that the time–aging time superposition can be applied for the PVA-based SPE (see Figure 6), we extrapolated the experimental data of relaxation modulus to one year and two years, respectively. As the $\sigma_{y}$ value of the PVA-LiClO_4_ specimen increased very slightly after an aging time of 20 days, the value at 30 days may be approximately regarded as the related yield stress ($\sigma_{y}=36.2$ MPa) for the long-term-aged SPE film. From Figure 8, $\left[h_{s}/h_{a}\right]$ is found as 0.2 for the PVA-LiClO4 film aged for one year. Although the critical value needed to be further amplified with prolonged aging time, it implies that the aging-induced mechanical damage of the SPE in an ASSB may be eliminated by properly designing the electrolyte thickness.

### 4.4. Impact of Electrochemical Loading Rate

**Figure 10:**
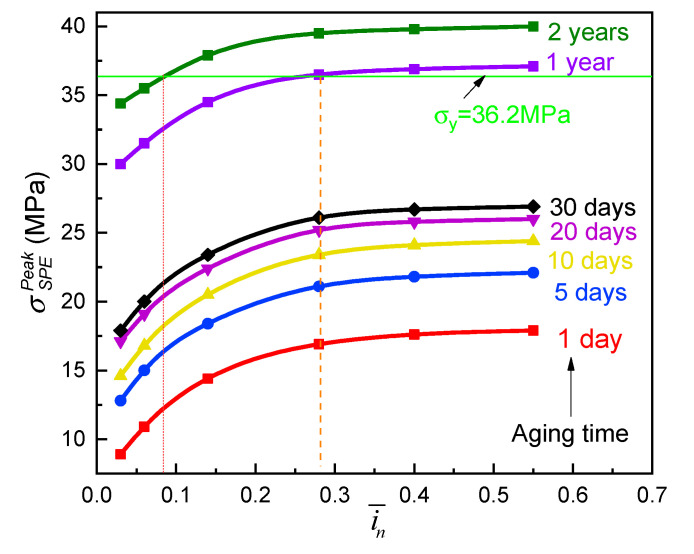
Effect of dimensionless surface current density (${\bar{i}}_{n}$) on the peak tensile stress.

Similarly, to investigate the influence role of the electrochemical condition on the mechanical integrity of SPE upon aging, the peak tensile stresses of SPE films for an ASSB with respect to the current density at various aging periods are also tracked and plotted in Figure 10. To ensure that the results are applicable to a wide range of operations, the dimensionless surface current density (${\bar{i}}_{n}$), which involves the current density ($i_{n}$), the thickness of the active layer ($h_{a}$), the lithium diffusion coefficient (D), as well as the stoichiometric maximum concentration of lithium ($c_{\max}$) based on the analytical work, were applied [53], i.e., ${\bar{i}}_{n}=i_{n}h_{a}/\left(FDc_{\max}\right)$. From Figure 9, for each aging SPE film with a relative thickness ratio of 0.2, the peak stress revealed a non-linear enlargement in the process of the lithiation. Initially, the value of $\sigma_{SPE}^{Peak}$ clearly boosted as the dimensionless parameter ${\bar{i}}_{n}$ raised to about 0.3, and then continued to grow toward a plateau slowly. Meanwhile, a distinct increase in the SPE stress resulting from prolonged aging could also be observed, regardless of the electrochemical loading rate. The reason behind this phenomenon may be ascribed to the fact that the influence of modulus relaxation caused by the discharging/charging operation is far less compared to the aging-induced stiffening effect, as the prior elapsed time is much longer than the lithiation process. It is worth noting that this tensile stress in the SPE film for an ASSB at ${\bar{i}}_{n}=0.28$ extended by at least 1.5, 2.1, and 2.3 times, reaching 26.1, 36.5, and 39.5 MPa, respectively, when the physical aging advanced from 1 to 30 days, one year, and two years. This means that a relatively higher lithiation rate ($\bar{i_{n}}\geq 0.28$) would cause yielding for the one-year-aged PVA-LiClO_4_, and the two-years-aged SPE could not maintain the structure integrity unless the critical current density further diminished to below 0.08. That is to say, decreasing the operation current can significantly improve the resistance to the mechanical failure in the aging SPE film.

As stated above, the mechanical completeness of SPE films in an ASSB is closely related to the surface current density and thickness ratio of the electrolyte to the electrode. In order to demonstrate the combined effects of the two important factors, and also obtain the optimized values for engineering purpose, a set of simulations were accomplished with the results depicted in Figure 10.

**Figure 11:**
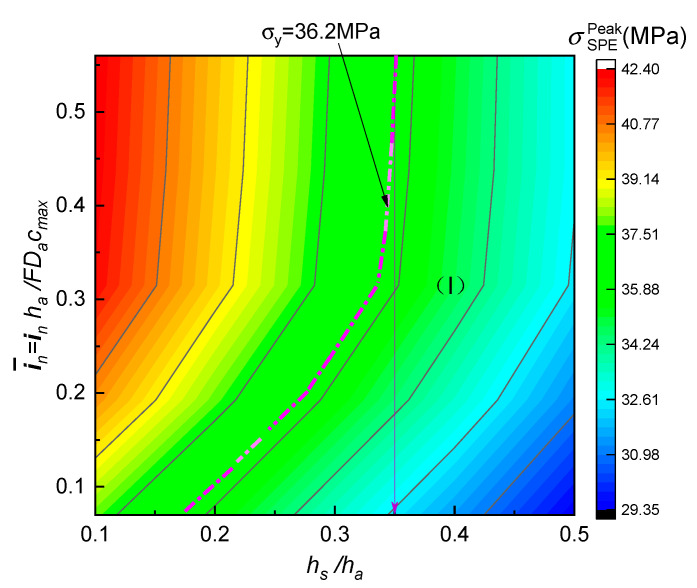
Combined effect of dimensionless surface current density (${\bar{i}}_{n}$) and thickness ratio ($h_{s}/h_{a}$) on the peak tensile stress of two-years-aged SPE film.

Figure 11 further verifies the coupled dependence of parameters ${\bar{i}}_{n}$ and $h_{s}/h_{a}$ on peak tensile stress in SPEs during the cell discharging operations. It can be seen that increasing the relative thickness of the electrolyte film resulted in a larger stress magnitude. As enhancing ${\bar{i}}_{n}$ significantly shortened the relaxation extent of the SPE elastic modulus, a greater impact was exhibited on this stress. With the help of the obtained yield stress ($\sigma_{y}=36.2$ MPa) of the electrolyte composite, the above 2D map is divided into two regions by a magenta curve, i.e., a safe region I that reaches the resistance to yield failure of the PVA-LiClO_4_ can be obtained as depicted in the right area of Figure 11. It is important to find that the two-years-aged SPE could void the mechanical damage under the normal lithiation condition where the discharging rate is less than 2.0 for an ASSB, i.e., ${\bar{i}}_{n}\leq 0.5$, if the thickness ratio of the electrolyte to the electrode is kept above 0.355. These results have provided essential insight into how to control the integrity degradation of the aging electrolyte material during electrochemical operation. It should be noted that the addition deformation generated by anion and cation diffusion has not been taken into account in the present investigation, due to the lack of necessary material constants such as the partial molar volume of solute (Ω) and stoichiometric maximum concentration ($C_{\max}$) for PVA-LiClO_4_. However, the ion migration-induced strain of the electrolyte actually alleviates the tensile stress in the SPE film for an ASSB at the discharging state. In addition, the nature and concentration of lithium salts in solid electrolyte and operation temperature can also affect the SPE durability. Although one may expect that a higher lithium salt content and lithiation temperature cannot only improve the ionic conductivity of the SPE but also cut down the mechanical stress due to the softening or plasticization effect, it accordingly weakens the electrolyte strength and speeds up the physical aging. Similarly, the addition of a small-molecule plasticizer such as dibutyl phthalate (DBP) and ionic liquid is also confronted with the same challenge. Moreover, the ionic conduction and mechanical strength of the polymer electrolyte materials SPE may be enhanced by crosslinking the polymer or introducing nonorganic fillers. However, it will increase the elastic modulus because of the hardening effect, giving rise to enlarging the peak tensile stress of the electrolyte. Actually, the interface between the SPE film and the electrode is not a perfectly bonding structure, and the contact area will decrease during the physical aging. Therefore, the effect of the above important factors on the mechanical behavior of SPEs should be considered in future research.

The interfacial stability between the solid electrolyte and electrode is a critical requisite for aging-resistant secondary lithium polymer batteries. It may be effectively improved by (1) adding nano-size TiO_2_, Al_2_O_3_, or SiO_2_ particles [54,55,56]; (2) the dispersion of ferroelectric microparticles (BaTiO_3_, LiNbO_3_, PbTiO_3_ [57], or g-LiAlO_2_ [58]); or (3) constructing a semi-interpenetrating polymer networks (s-IPN) structure [59]. However, it is still unclear how these modified measures affect the mechanical integrity degradation of SPEs in a lithium cell during calendar aging; one should have paid close attention to the issue.

## 5. Conclusions

(1) The PVA-based SPE films exhibited a pronounced physical aging trend under ambient storage conditions, in which both stiffness and yield strength enhanced with the elapsed time. However, the tensile rupture stress nearly did not rely on the aging time, due to the mechanical rejuvenation in the process of plastic deformation.
(2) The KWW time-decay function could describe the evolution of elastic modulus for aging SPEs during the stress relaxation period. Furthermore, it is found that the physical aging contributed to the increase in initial modulus and characteristic relaxation time, while the shape factor remained constant for the specimen at different aging stages. Accordingly, the ideal momentary relaxation master curve could be obtained using the classical Struik shift method in terms of the time–aging time superposition.
(3) The peak tensile stress in the SPE film occurred at the electrolyte/cathode interface for a full discharged ASSB, it would significantly enlarge with the aging on account of the stiffening of the electrolyte composite easily resulting in the mechanical failure of the cell system. However this negative effect may be restrained by increasing the relative thickness of the solid electrolyte to the composite electrode. In addition a lower rate discharge is a benefit of the durability of the SPE during physical aging.
(4) In order to meet the requirement of a two-year lifetime needed for potential commercialization, the relative thickness of the electrolyte to the electrode should be larger than 0.355 for the ASSB of the Li/PVA-40% LiClO_4_/LiCoO_2_ in the viewpoint of mechanics.

## Figures and Tables

**Table 1:** Sets of material parameters used in simulation.

	SPE (PVA-40% LiClO_4_)	Cathode (LiCoO_2_:PVdF:CB(Carbon Black) = 85:10:5)	Current Collector (Al)
Elastic modulus, *E* (GPa)	Indicated in Figure 4	1.97 *	70
Poisson ratio, *μ*	0.3	0.31 *	0.3
Partial molar volume of solute Ω (m^3^·mol^−1^)	/	4.17 × 10^–^^6^ [50]	/
Diffusion coefficient, *D* (m^2^·s^−1^)	/	1.76 × 10^–^^15^ [51]	/
Stoichiometric maximum concentration, *c*_max_ (mol m^−3^)	/	2.33 × 10^4^ [51]	/
Thickness, *h* (μm)	5	10	10

* The elastic modulus and Poisson ratio of composite cathode determined by the S-combining rule [52] can be seen in Supplementary Materials.

## Supplementary Materials

The following are available online at https://www.mdpi.com/2073-4360/12/9/1886/s1, Table S1: Material properties of the constituents of LiCoO_2_ cathode composite.
