# Supplementary material for: Mechanical Integrity Degradation and Control of All-Solid-State Lithium Battery with Physical Aging Poly (Vinyl Alcohol)-Based Electrolyte
Source: Polymers (Basel). 2020 Aug 21;12(9):1886. doi: 10.3390/polym12091886 (PMC7565167; doi:10.3390/polym12091886)
Supplement: Supplementary file 1 [file polymers-12-01886-s001.pdf]

Calculation method for the equivalent elastic constants of $LiCoO_2$ electrode composites

As well documented in the literature, the $LiCoO_2$ cathode coating is a typical composite with $LiCoO_2$ particles, conductive carbon black (CB), PVdF binder and pores. Based on the reported material properties of the constituents in $LiCoO_2$ electrode coating listed in Table S1, one can estimate its equivalent elastic properties (Young's modulus and Poison's ratio) as following method.

Table S1 Material properties of the constituents of $LiCoO_2$ cathode composite

	$LiCoO_2$	PVdF	Carbon Black
ρ ($g \cdot cm^{-3}$)	5.1 [1]	1.78 [2]	1.9 [3]
E (GPa)	170 [4]	2.0 [2]	32.47 [3]
ν	0.3 [2]	0.34 [2]	0.315 [3]
The mass content (%) [5]	85.0	10.0	5.0

Firstly, the lower bounds of the effective shear modulus (G_m) and bulk modulus (K_m) of a matrix containing CB particles and PVdF binder are determined using the inverse rule of mixtures.

$$\frac{1}{K_m} = \frac{\theta_{m,\mathrm{CB}}}{K_{CB}} + \frac{\theta_{m,\mathrm{PVdF}}}{K_{PVdF}} \qquad \text{(S1a)}$$

$$\frac{1}{G_m} = \frac{\theta_{m,\mathrm{CB}}}{G_{CB}} + \frac{\theta_{m,\mathrm{PVdF}}}{G_{PVdF}} \qquad \text{(S1b)}$$

$$\theta_{m,\mathrm{PVdF}} = \frac{\theta_{PVdF}}{\theta_{CB} + \theta_{PVdF}} \qquad \text{(S1c)}$$

$$\theta_{m,\mathrm{CB}} = \frac{\theta_{CB}}{\theta_{CB} + \theta_{PVdF}} \qquad \text{(S1c)}$$

where K_{CB} and K_{PVdF} are the bulk modulus of the CB particles and PVdF binder

respectively, while G_{CB} and G_{PVdF} are the corresponding shear modulus. $\theta_{m,\mathrm{CB}}$ and $\theta_{m,\mathrm{PVdF}}$ are the volume fractions of the CB and the PVdF in the CB/PVdF matrix, respectively. In addition, θ_{CB} and θ_{PVdF} are the volume fractions of the CB and the PVdF in the $LiCoO_2$ composite, respectively.

Secondly, in order to consider the effect of the porosity of 30% in LiCoO2 composite, open cell theory for an isotropic porous solid is adapted to evaluating the effective bulk modulus of a porous matrix (K_{pm}) with the previously defined CB and PVdF matrix as the solid part, and is given by Equation (S2a-c).

$$K_{pm} = \frac{1}{3\left(1-2\nu_{pm}\right)} \frac{9K_m G_m}{3K_m + G_m} \left(\frac{\rho_{pm}}{\rho_m}\right)^2 \tag{S2a}$$

with

$$\begin{aligned} \rho_m &= \theta_{m,\mathrm{CB}} \rho_{CB} + \theta_{m,\mathrm{PVdF}} \rho_{PVdF} \\ \rho_{pm} &= \theta_{pm,\mathrm{CB}} \rho_{CB} + \theta_{pm,\mathrm{PVdF}} \rho_{PVdF} \end{aligned} \tag{S2b}$$

and

$$\begin{aligned} \theta_{pm,\mathrm{CB}} &= \frac{\theta_{CB}}{\theta_{pm}} \\ \theta_{pm,\mathrm{PVdF}} &= \frac{\theta_{PVdF}}{\theta_{pm}} \\ \theta_{pm} &= \theta_{CB} + \theta_{PVdF} + \theta_P = 1 - \theta_A \end{aligned} \tag{S2c}$$

where ρ_m is the density the solid portion of the CB/PVdF matrix, ρ_{pm} and θ_{pm} are the density and volume fraction of the porous matrix (CB, PVdF and porosity), respectively. Meanwhile, $\theta_{pm,\mathrm{CB}}$ and $\theta_{pm,\mathrm{PVdF}}$ are the volume fractions of CB and PVdF in the CB/PVdF porous matrix, respectively. Besides, θ_P and θ_A are the volume fractions of porosity and active particles of the $LiCoO_2$ composite, respectively. The Poisson's ratio of the porous matrix (ν_{pm}), is assumed to be equal to 1/3.

Thirdly, according to 'S-combining rule' [3], the bulk modulus (K_e) and shear

modulus (G_e) of the $LiCoO_2$ composite are identified by Equations (S3a-d) and (S4a-d) as followed.

$$K_e = \frac{K_{pm}\left(1+\theta_A \xi_l \chi\right)}{1-\theta_A \Psi \chi} \tag{S3a}$$

with

$$\chi = \frac{K_A - K_{pm}}{K_A + \xi_l K_{pm}} \tag{S3b}$$

$$\Psi = 1 + \frac{\theta_A \theta_{pm}\left(1-\frac{1}{2}\theta_{pm}\right)\left(K_A - K_{pm}\right)\left(\xi_u - \xi_l\right)}{K_A + \xi_u\left(\theta_A K_A + \theta_{pm} K_{pm}\right)} \tag{S3c}$$

$$\xi_u = \frac{2\left(1-2\nu_A\right)K_A}{\left(1+\nu_A\right)K_{pm}} \qquad \xi_l = \frac{2\left(1-2\nu_{pm}\right)}{\left(1+\nu_{pm}\right)} \tag{S3d}$$

where ν_A and K_A are the Poisson's ratio and bulk modulus of $LiCoO_2$ particles, respectively.

$$G_e = \frac{G_{pm}\left(1+\theta_A \xi_{Al} \chi_A\right)}{1-\theta_A \Psi_A \chi_A} \tag{S4a}$$

with

$$\chi_A = \frac{G_A - G_{pm}}{G_A + \xi_{Al} G_{pm}} \tag{S4b}$$

$$\Psi_A = 1 + \frac{\theta_A \theta_{pm}\left(1-\frac{1}{2}\theta_{pm}\right)\left(G_A - G_{pm}\right)\left(\xi_{Au} - \xi_{Al}\right)}{G_A + \xi_{Au}\left(\theta_A G_A + \theta_{pm} G_{pm}\right)} \tag{S4c}$$

$$\xi_{Au} = \frac{\left(7-5\nu_A\right)G_A}{\left(8-10\nu_A\right)G_{pm}} \qquad \xi_{Al} = \frac{7-5\nu_{pm}}{8-10\nu_{pm}} \tag{S4d}$$

where G_A is the shear modulus of $LiCoO_2$ particles.

Finally, the Young's modulus and Poison's ratio of electrode coating composite have been acquired in terms of the following equation, respectively.

$$K_e = \frac{E_e}{3\left(1-2\nu_e\right)} \tag{S5a}$$

$$G_e = \frac{E_e}{2\left(1+\nu_e\right)} \tag{S5b}$$

Solving above Equation (S5a) and (S5b), we can obtain the equivalent elastic constants of $LiCoO_2$ cathode composite, i.e. $E_e = 1.97\text{GPa}, \nu_e = 0.31$

Reference

[1] Oudenhoven Jos F.M.; Baggetto Loïc; Notten Peter H.L. All-solid-state lithium-ion microbatteries: A review of various three-dimensional concepts. *Adv. Energy Mater.*, **2011**, 1(1), 10-33.

[2] Qi Y.; Hector Jr L.G; James C.; et al. Lithium concentration dependent elastic properties of battery electrode materials from first principles calculations. *J. Electrochem. Soc.*, **2014**, 161(11), F3010-F3018.

[3] Jones E.M.C; Silberstein M.N.; White S.R.; et al. In situ measurements of strains in composite battery electrodes during electrochemical cycling. *Exp. Mech.*, **2014**, 54(6), 971-985.

[4] Noman I.; Lee S. Mechanical failure analysis of graphite anode particles with PVDF binders in Li-ion batteries. *J. Electrochem. Soc.*, **2018**, 165(9), A1961-A1970.

[5] Wang Z.X.; Li L.J; Chen L.Q.; Huang X.J. Structural and electrochemical characterizations of surface-modified $LiCoO_2$ cathode materials for Li-ion batteries. *Solid State Ionics*, **2002**, 148(3-4), 335-342.
